# Personality traits are not associated with changes in employment status over 3 years in persons with multiple sclerosis

**DOI:** 10.1177/20552173221145576

**Published:** 2022-12-19

**Authors:** K van der Hiele, EEA van Egmond, DAM van Gorp, PJ Jongen, MF Reneman, JJL van der Klink, EAC Beenakker, JJJ van Eijk, STFM Frequin, E Hoitsma, OHH Gerlach, JP Mostert, WIM Verhagen, MAP Heerings, HAM Middelkoop, LH Visser

**Affiliations:** Institute of Psychology, Health, Medical and Neuropsychology Unit, 4496 Leiden University, Leiden, The Netherlands; Institute of Psychology, Health, Medical and Neuropsychology Unit, 4496 Leiden University, Leiden, The Netherlands; Department of Care Ethics, University of Humanistic Studies, Utrecht, The Netherlands; National Multiple Sclerosis Foundation, Rotterdam, The Netherlands; Department of Neurology, 7898 Elisabeth-TweeSteden Hospital, Tilburg, The Netherlands; Department of Care Ethics, University of Humanistic Studies, Utrecht, The Netherlands; MS4 Research Institute, Nijmegen, The Netherlands; Department of Community & Occupational Medicine, University of Groningen, University Medical Centre Groningen, Groningen, The Netherlands; Department of Rehabilitation Medicine, Centre for Rehabilitation, University of Groningen, University Medical Centre Groningen, Haren, The Netherlands; Department of Social and Behavioural Sciences, Tranzo Scientific Centre for Care and Welfare, 7899 Tilburg University, Tilburg, The Netherlands; Optentia, North West University of South Africa, Vanderbijlspark, South Africa; Department of Neurology, 4480 Medical Centre Leeuwarden, Leeuwarden, The Netherlands; Department of Neurology, Jeroen Bosch Hospital, ‘s-Hertogenbosch, The Netherlands; Department of Neurology, St Antonius Hospital, Nieuwegein, The Netherlands; Department of Neurology, Alrijne Hospital Leiden, Leiden, The Netherlands; Department of Neurology, Zuyderland Medical Centre, Sittard-Geleen, The Netherlands; Department of Neurology, Rijnstate Hospital, Arnhem, The Netherlands; Department of Neurology, 6030 Canisius-Wilhelmina Hospital, Nijmegen, The Netherlands; Department of Human Genetics, Radboud University Medical Centre, Donders Institute for Brain, Cognition and Behaviour, Nijmegen, The Netherlands; Institute of Psychology, Health, Medical and Neuropsychology Unit, 4496 Leiden University, Leiden, The Netherlands; Department of Neurology & Neuropsychology, Leiden University Medical Centre, Leiden, The Netherlands; Department of Care Ethics, University of Humanistic Studies, Utrecht, The Netherlands; Department of Neurology, 7898 Elisabeth-TweeSteden Hospital, Tilburg, The Netherlands

**Keywords:** Multiple sclerosis, employment status, personality, mood, fatigue, prospective study

## Abstract

Previous research discovered a protective effect of higher conscientiousness against a 3-year deterioration in employment status in persons with multiple sclerosis (pwMS). To replicate these findings, we used data from a multicentre prospective cohort study where 145 employed pwMS completed questionnaires, neurological and neuropsychological examinations at baseline and after 3 years. A 3-year deterioration in employment status was reported in 31.0%. We observed no differences in personality, demographics or clinical characteristics between pwMS with deteriorated or stable employment status. These null findings may be partly explained by the classification of deteriorated employment status, which does not reflect Dutch labour conditions.

## Introduction

Personality traits have been reported as protective factors for job loss in persons with multiple sclerosis (pwMS). Studies reported higher conscientiousness^1,2^ and agreeableness^3^ in employed as compared to unemployed persons with MS, after accounting for confounding factors such as disability, cognition, mood and fatigue. In contrast, we found no associations between personality traits and self-reported occupational functioning in pwMS when correcting for similar confounders in a cross-sectional study.^4^ Yet a recent prospective study Jaworski et al.,^5^ based on a US population, observed a protective effect of higher conscientiousness against a deterioration in employment status 3 years later. More prospective research is clearly needed, and is of interest because the behavioural characteristics associated with personality traits are potentially modifiable.^6^ The current study aimed to replicate and extend the findings by Jaworski et al.^5^ We examined whether personality traits, including all big-five personality traits,^7^ are predictive of 3-year employment status in pwMS.

## Methods

This prospective cohort study included 145 pwMS (relapsing-remitting subtype) from the MS@Work study.^8^ We included participants who were gainfully employed at baseline, did not reach the retirement age during the study, were not self-employed, and completed the necessary questionnaires at baseline and 3-year follow up. Participants underwent yearly neurological and neuropsychological examinations, and completed yearly online questionnaires.

Employment status and negative work events were classified based on Jaworski et al.^5^: (a) employed full-time, working 25 to 40 h per week; (b) employed full-time with reduced hours or responsibilities; (c) employed part-time, <25 h per week; (d) unemployed, able bodied but unable to find a job; (e) unemployed, receiving disability benefits; (f) unemployed, unable to work but not receiving benefits. Participants who worked no hours or part-time because of (partial) sickness absence were categorised as (c). Participants were considered to have a ‘deteriorated employment status’ (DES) if they reported either a deterioration in employment classification (e.g. full-time to part-time) or more negative work events from baseline to 3-year follow up. Participants who reported no changes or had increased their employment classification were classified as having a ‘stable employment status’ (SES).

We first examined differences between DES and SES using parametric and non-parametric tests where applicable. A multivariate logistic regression analysis was planned to examine predictors of 3-year employment status, including variables differing significantly between groups (*p* *<* 0.05). Ethical approval was obtained (METC Brabant: NL43098.008.12 1307).

## Results

**Figure 1.**
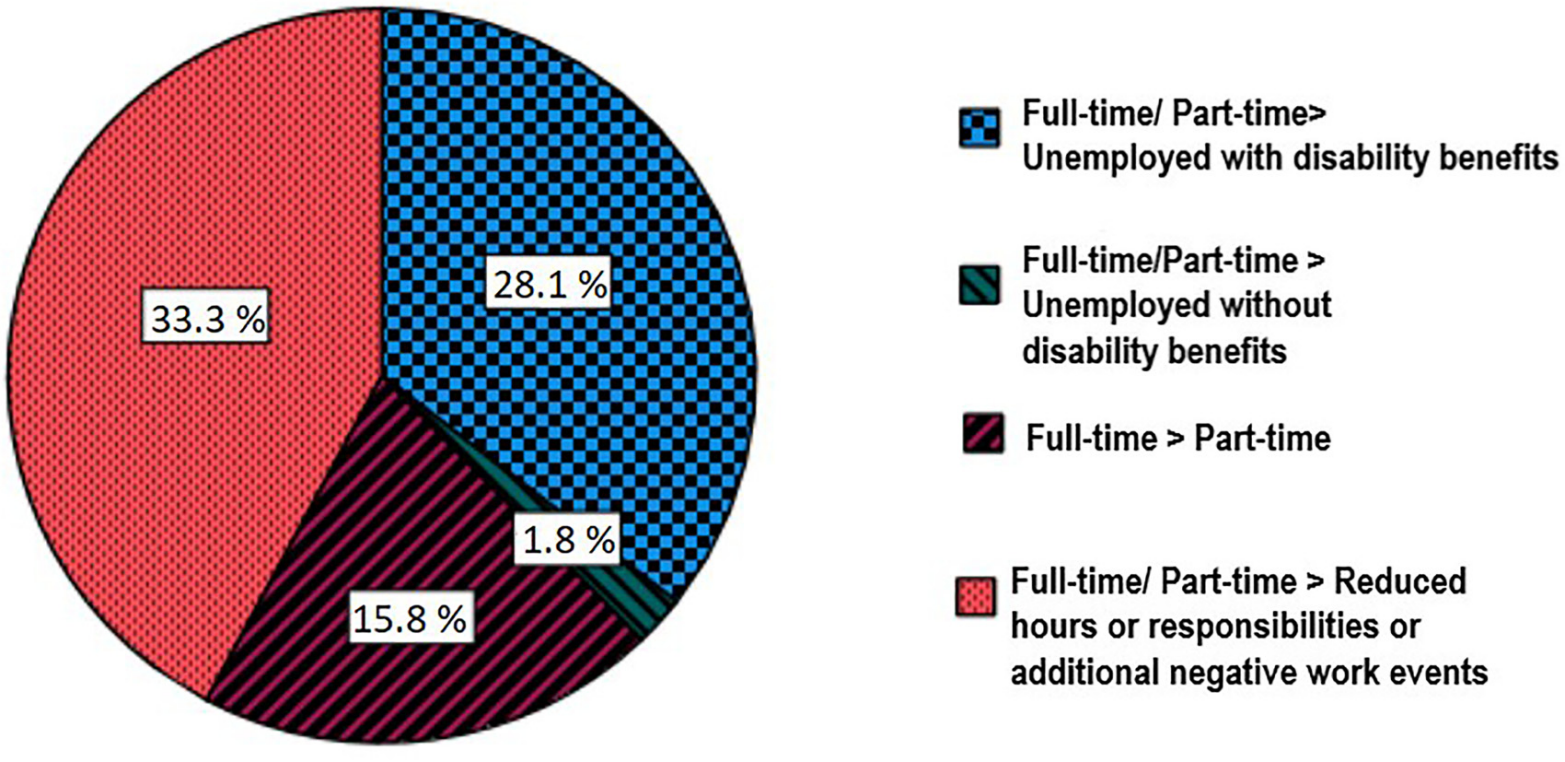
Specification of deterioration in employment status at 3-year follow up.

At baseline, 52.4% were employed part-time, 40.0% full-time and 7.6% full-time with reduced responsibilities. DES was reported by 31.0% of the pwMS. A further specification is presented in Figure 1. No baseline differences in personality traits, demographics or clinical characteristics were observed between DES and SES (Table 1). Consequently, the planned multivariate logistic regression analysis was not performed.

**Table 1.** Baseline demographic and clinical characteristics for persons with multiple sclerosis with DES and SES at 3-year follow up.

	DES N = 45		SES N = 100		*p value*
	*Mean/* *Median/* *N*	*SD/* *IQR/* *%*	*Mean/* *Median/* *N*	*SD/* *IQR/* *%*	
Female^a^	38	84.4%	80	80.0%	.349
Age^b^	42.0	9.7	41.5	8.8	.771
Educational level^a^					
Low	7	15.6%	10	10.0%	.609
Medium	17	37.8%	38	38.0%	
High	21	46.7%	52	52.0%	
Disease duration (years)^c^	5.0	9.0	5.0	8.0	.949
Physical disability (EDSS; 0–10)^c^	2.0	1.5	2.0	1.5	.064
Fatigue (MFIS; 0–84)^b^	33.4	13.6	33.1	14.8	.845
Depression (HADS depression; 0–21)^c^	2.0	4.0	2.0	3.0	.577
Anxiety (HADS anxiety; 0–21)^c^	4.0	3.0	5.0	5.0	.169
Information processing speed (SDMT; 0–110)^b^	53.1	8.6	55.7	8.0	.084
Neuroticism (NEO-FFI; 12–60)^b^	26.8	7.0	28.0	7.4	.329
Extraversion (NEO-FFI; 12–60)^b^	41.6	6.5	41.9	5.6	.684
Openness (NEO-FFI;12–60)^b^	37.4	7.1	35.9	6.0	.196
Agreeableness (NEO-FFI;12–60)^c^	46.0	5.0	46.0	5.0	.981
Conscientiousness (NEO-FFI;12–60)^b^	46.3	5.0	47.3	5.5	.324

^a^ N(%).

^b^ Mean (SD).

^c^ Median (IQR).

Educational level: low = finished low-level secondary school, middle = finished secondary school at a medium level and high = finished secondary school at the highest level and/or obtained a college/university degree.

DES: deteriorated employment status; EDSS: Expanded Disability Status Scale score; HADS: Hospital Anxiety and Depression Scale scores per domain; IQR: interquartile range; MFIS: Modified Fatigue Impact Scale (total impact of fatigue); NEO-FFI: Neo Five Factory Personality Inventory; SD: standard deviation; SDMT: Symbol Digit Modalities Test (total correct); SES: stable employment status.

## Discussion

Neither personality, nor demographic or clinical characteristics were associated with a 3-year deterioration in employment status. This was unexpected, because strong evidence exists for age, disease duration, mobility and fatigue as predictors of future work-related difficulties.^9^ In the study by Jaworski et al.,^5^ the personality trait conscientiousness was the sole significant predictor of DES, explaining 52.5% of variance. Their model corrected for education, race, fatigue, visuospatial memory, cognitive processing speed and manual dexterity speed, but no other personality traits were examined. We will provide an explanation for the null findings in the current study, advocate to study predictors focusing on the individual within their social context, and to reconsider the DES/SES classification method.

The percentage of pwMS reporting DES in the current study (31.0%) is roughly similar to that in the Jaworski study (25.7%).^5^ However, the reasons for DES differ greatly. While in the Jaworski study most workers with DES reported unemployment after 3 years (61.0%), the main reason for DES in our sample was a reduction in work hours/ responsibilities or additional negative work events (33.3%). Our lower percentage of 3-year unemployment may be related to the fact that our MS group is less disabled and has a shorter disease duration. Furthermore, most pwMS worked part-time at baseline (52.0%). In the Netherlands, part-time work is very common and acceptable, with 18% of the working population working <20 h per week (CBS StatLine; second quarter 2021). When working part-time, it may be easier to maintain work–life balance when faced with MS-related work difficulties. Another important difference with the Jaworski study is that the pwMS in our sample reported little negative work events (79.3% reported no negative work events), many used work accommodations (72.4% used one or more accommodations) and most disclosed their MS diagnosis (98.5% disclosure). Dutch labour legislation, especially the Gatekeeper Act of 2002 might have contributed to availability and access to accommodations including (temporary) part-time work, and a safer environment, leading to more disclosure, at least for the (highly educated) pwMS in the current study. Perhaps personality traits and clinical factors are less relevant predictors of DES/SES in a group of mildly disabled pwMS who already made important work adaptations, for example, by working part-time, disclosing disease status and by making use of work accommodations. Further research is necessary to find relevant predictors of DES/SES in order to prevent premature job loss in MS, especially in earlier disease stages. Instead of only examining individual (e.g. psychological and clinical) predictors, we suggest focusing on the individual within their social context, for example by taking into account the inclusiveness of the workplace, labour legislation, balance between job resources and demands, and work–home balance.

We should reconsider the DES/SES classification method. The current classification considers reducing work hours or responsibilities as a negative development, while such reductions may also be viewed as a successful manner to cope with work difficulties arising due to MS, especially when agreed upon with the employer. The currently used DES/SES classification may not be applicable to the Dutch situation, where part-time work is common and acceptable and may reflect a deliberate choice. It is of interest to identify other objective and subjective ways of classifying DES/SES, wherein a deterioration truly reflects a decrease in quality of working life.
